# Yeast Irc22 Is a Novel Dsk2-Interacting Protein that Is Involved in Salt Tolerance

**DOI:** 10.3390/cells3020180

**Published:** 2014-03-27

**Authors:** Takashi Ishii, Minoru Funakoshi, Hideki Kobayashi, Takeshi Sekiguchi

**Affiliations:** 1 Research Center for Control of Aging, Fukuoka Dental College, Tamura 2-15-1, Sawara-ku, Fukuoka 814-0193, Japan; 2 Department of Molecular Biology, Graduate School of Medical Sciences, Kyushu University, Maidashi 3-1-1, Higashi-ku, Fukuoka 812-8582, Japan; E-Mails: funakoshi@fucoidan1.jp (M.F.); sekigu@molbiol.med.kyushu-u.ac.jp (T.S.); 3 Center for Faculty Development, Okayama University, Tsushima-naka 2-1-1, Kita-ku, Okayama 700-8530, Japan

**Keywords:** UBL-UBA protein, Dsk2, Irc22/*YEL001C*, ubiquitin-dependent protein degradation, salt tolerance, budding yeast

## Abstract

The yeast ubiquitin-like and ubiquitin-associated protein Dsk2 is one of the ubiquitin receptors that function in the ubiquitin-proteasome pathway. We screened the Dsk2-interacting proteins in *Saccharomyces cerevisiae* by a two-hybrid assay and identified a novel Dsk2-interacting protein, Irc22, the gene locus of which has previously been described as *YEL001C*, but the function of which is unknown. *IRC22*/*YEL001C* encodes 225 amino acid residues with a calculated molecular weight of 25 kDa. The Irc22 protein was detected in yeast cells. *IRC22* was a nonessential gene for yeast growth, and its homologs were found among ascomycetous yeasts. Irc22 interacted with Dsk2 in yeast cells, but not with Rad23 and Ddi1. Ubiquitin-dependent degradation was impaired mildly by over-expression or disruption of *IRC22.* Compared with the wild-type strain, *dsk2Δ* exhibited salt sensitivity while *irc22Δ* exhibited salt tolerance at high temperatures. The salt-tolerant phenotype that was observed in *irc22Δ* disappeared in the *dsk2Δirc22Δ* double disruptant, indicating that *DSK2* is positively and *IRC22* is negatively involved in salt stress tolerance. *IRC22* disruption did not affect any responses to DNA damage and oxidative stress when comparing the *irc22Δ* and wild-type strains. Collectively, these results suggest that Dsk2 and Irc22 are involved in salt stress tolerance in yeast.

## 1. Introduction

The ubiquitin-proteasome pathway plays a crucial role in the control of various cellular processes by mediating the degradation of many short-lived proteins. Proteins that are targeted for degradation are ubiquitinated by a cascade of ubiquitin-activating (E1), ubiquitin-conjugating (E2), and ubiquitin-ligating (E3) enzymatic reactions. Polyubiquitinated proteins are then recognized by ubiquitin receptors and degraded by the proteasome (see reviews [1,2]). Ubiquitin-like and ubiquitin-associated (UBL-UBA) proteins act as shuttling ubiquitin receptors that bind to polyubiquitinated proteins and deliver them to proteasomal ubiquitin receptors on the regulatory particles of the 26S proteasome (see reviews [3,4,5,6]). UBL-UBA proteins including Rad23, Dsk2, and Ddi1 contain a UBL domain at the N-terminus and a UBA domain at the C-terminus. The C-terminal UBA domain binds polyubiquitin chains and the N-terminal UBL domain binds to a docking site of the proteasome [7,8,9]. 

UBL-UBA proteins interact and cooperate with the proteasomal ubiquitin receptors Rpn1, Rpn10, and Rpn13 [10,11,12,13,14,15], E1/E2/E3 enzymes [16,17,18], and several accessory proteins [19,20,21,22]. Those interactions are positively and negatively involved in the recognition and regulation of the ubiquitin-proteasome pathway: Via their UBL domains, Rad23 and Dsk2 interact with the ubiquitin ligase E4 (Ufd2) that is defined as a ubiquitin chain-extending activity, and the Rad23-Ufd2 interaction helps to recognize ubiquitinated substrates [19,22]. Rpn10 competes for Dsk2 binding with the proteasome [23], and Dsk2, via its UBL domain, interacts with Rpn10 depending on length of polyubiquitin chains that serve as an ubiquitin-chain sensor on the proteasome in the delivery of the degradable proteins [24]. Rad23 and Dsk2, via their UBL domains, also interact with peptidyl-tRNA hydrolase 2 (Pth2), and the UBL domain-Pth2 interaction may regulate the release of the UBL-UBA protein from the proteasome [21]. The C-terminal UBA domain protects Rad23 and Dsk2 shuttle receptors from degradation by the proteasome [25]. In response to endoplasmic reticulum (ER) stress, Cdc48, along with Dsk2 and Rad23, participates in ER-associated degradation (ERAD) [20] through the interaction of the UBL domain with Ufd2 [22].

UBL-UBA proteins display a functional redundancy in the ubiquitin-proteasome pathway. None of the UBL-UBA proteins are essential for viability. For instance, *dsk2Δ* and *rad23Δ* single deletions are viable and have little effect on protein degradation while the double deletion *dsk2Δrad23Δ* is toxic and stabilizes degradation completely [26,27]. *RAD23* is redundant with *DSK2* and *DDI1,* and the triple deletion *rad23Δdsk2Δddi1Δ* shows a synthetic effect [28]. Physical interactions are also redundant among UBL-UBA proteins. Different members of the UBL-UBA protein family form homo- and heterodimers that are implicated in redundant functions of UBL-UBA proteins [29,30,31,32,33,34]. Furthermore, docking sites for Rad23 and Dsk2 on a regulatory particle of the proteasome are shown to be divergent in different species [13]. In yeast, Rad23 and Dsk2 prefer Rpn1 and Rpn10, respectively, as a docking partner. In mammals, in contrast, Rad23 and Dsk2 interact with both Rpn10 and Rpn13. Together, diverse and redundant properties of the UBL-UBA protein family seem to be involved in substrate selectivity and some distinct roles in the ubiquitin-proteasome pathway.

Besides the UBA and UBL domains, UBL-UBA proteins also contain a variety of characteristic conserved sequences in the middle stretch; these domains/motifs seem to facilitate proteasomal degradation and/or play distinct cellular roles. For instance, the DNA repair protein Rad4 binds to the *xeroderma pigmentosum* group C (XPC)-binding domain of Rad23 and cooperates not only in nucleotide excision repair of DNA damage response but also in protein degradation [35,36]. The retroviral aspartyl-protease domain of Ddi1 is required for both its dimerization and the Pds1 activity in cell cycle control [37,38]. Dsk2 contains the stress-inducible, heat shock chaperonin-binding motifs (STI1 motifs) that are required for binding to the HSP70 family protein Stch [39]. In response to ER stress, Dsk2, together with Rad23, helps to recruit the misfolding proteins accumulated in the ER to the proteasome [20], which implies the involvement of the Dsk2 chaperone functions. Furthermore, the STI1 motifs of A1Up, a human paralog of Dsk2, interact with the small hydrophobic protein that plays a role in the process of viral infection [40]. Probing the interacting partners with UBL-UBA proteins would shed light on their unique roles in cellular functions including their non-proteasomal degradation.

In search for interacting proteins of Dsk2 in a two-hybrid screen, we identified Irc22/*YEL001C* in *Saccharomyces cerevisiae*, which has an unknown function. In this report, we describe a novel Dsk2-interacting protein, Irc22, that is relevant to ubiquitin-dependent protein degradation. Irc22 interacts with Dsk2 but not with Rad23 and Ddi1. Irc22 affects the ubiquitin-dependent degradation system. Moreover, Irc22, together with Dsk2, appears to participate in salt tolerance. A link between ubiquitin-dependent degradation and salt stress responses has been recently reported in plants: the proteasomal receptor Rpn10 [41], ubiquitin-conjugating enzyme E2 [42], ubiquitin ligase E3 [43], and ERAD components [42,44]. To date, however, an involvement of UBL-UBA proteins in salt stress responses has not been described. Here, we show that deletion of *DSK2* induces salt sensitivity while deletion of *IRC22* induces salt tolerance in yeast, thereby indicating that Dsk2 is positively and Irc22 is negatively involved in salt stress tolerance. Possible roles of Dsk2 and Irc22 in salt stress in yeast are discussed.

## 2. Experimental Section

### 2.1. Strains and Media

The yeast strains used in this study were in the *S. cerevisiae* YPH499 (*MAT***a** *ade2-101 leu2-Δ1 trp1-Δ63 ura3-52 lys2-801 his3-Δ200*) background [45]. Yeast strains were grown in yeast extract-peptone-dextrose (YPD) or selective medium. The yeast strain deleted for the *IRC22* gene was generated by PCR-mediated gene disruption [46] and verified by PCR and immunoblot analysis. For overexpression experiments, yeast cells transformed with a galactose-inducible plasmid were cultured in minimal medium containing 2% raffinose at 30 °C to OD_600_ = 0.8. Galactose (2%) then was added to the medium and the cells were incubated at 30 °C for 4 h. The *Escherichia coli* DH5a strain was used for DNA manipulation, and *E. coli* BL21 (DE3) was used for the expression of recombinant protein. The yeast strain Y190 (*MAT***a** *ade2-101 his3-Δ200 leu2-3,112 trp1-901 ura3-52 gal4Δ gal80Δ cyh^r^2 URA3::Gal-lacZ LYS2::GAL-HIS3*) and PJ69-4A (*MAT***a** *trp1-901 leu2-3,112 ura3-52 his3-Δ200 gal4Δ gal80Δ LYS2::GAL1-HIS3 GAL2-ADE2 met2::GAL7-lacZ*) were used for the two-hybrid screening. *DSK2* lacking the N terminus (72-373) was subcloned into pAS404 and used as bait in the two-hybrid screen using a yeast Gal4AD-cDNA library [8]. Transformants that grew on the SD-His-Trp-Leu +25 mM 3-aminotriazole plate were selected and the library clones were obtained and sequenced.

### 2.2. Plasmids and Mutants

For p*GAL1*-YEplac112-T7 vector construction, T7 tag module was generated with PCR and subcloned into downstream of *GAL1* promoter in p*GAL1*-YEplac112 vector using a *Nde*I-*Bam*H1 site. p*GAL1*-YEplac112 vector was generated from YEplac112 vector and *GAL1* promoter module derived from pMR438 vector using a *Eco*RI-*Bam*HI site. For the binding assays, the PCR-amplified *IRC22* open reading frame (ORF) was subcloned into a *Bam*HI-*Hin*dIII site in the galactose-inducible vector p*GAL1*-YEplac112-T7. For over-expression, *IRC22* DNA was subcloned into a *Bam*HI-*Hin*dIII site in the galactose-inducible vectors p*GAL1-*YEplac112, p*GAL1-*YEplac181, and p*GAL1-*YEplac195. For the expression of GST-fused Irc22, the PCR-amplified *IRC22* ORF was subcloned into an *Xba*I-*Hin*dIII site in the galactose-inducible vector pEG(KG). For the expression of His_6_-T7-tagged Irc22, *IRC22* DNA was subcloned into a *Bam*HI-*Hin*dIII site in the expression vector pET-28a(+). *IRC22* deletion mutants were constructed by the PCR method and subcloned into p*GAL1*-YEplac112-T7. The PCR-amplified *DSK2, RAD23, DDI1,* and *PTH2* ORFs were subcloned into a *Bam*HI-*Sal*I site in pEG(KG) and pGEX(KG) to construct GST-tagged Dsk2, Rad23, Ddi1, and Pth2. Plasmids carrying Ub-Leu-β-galactosidase (β-gal) were originally provided by Dr. A. Varshavsky.

### 2.3. Pull-Down Assays and Immunoblotting

Cell extracts were prepared using glass beads in lysis buffer: 20 mM HEPES, pH 7.5, 100 mM NaCl, 0.5% Nonidet P-40, 1 mM ethylene-diaminetetraacetic acid, 1 mM *p*-amidinophenylmethanesulfonyl fluoride, and 1 μg/mL each of leupeptin, pepstatin and chymostatin. For the GST pull-down assay, cell extracts (5.0 A_600_ cells equivalent) or recombinant proteins (1 mg) were incubated with 20 μL glutathione-Sepharose 4B (Pharmacia) in 500 μL lysis buffer for 1 h at 4 °C. The beads were washed three times with lysis buffer, followed by sodium dodecyl sulfate (SDS)-polyacrylamide gel electrophoresis of the retained proteins and immunoblotting with anti-GST. Cell extracts equivalent to 0.2 A_600_ were used for immunoblotting of cell extracts. Recombinant GST-fused proteins and His_6_-tagged proteins were prepared from *E. coli* BL21 (DE3) transformed with expression vectors. 

### 2.4. Degradation Assay of N-End Rule Substrate

The plasmid carrying the Ub-lacZ fusion gene [47] was transformed into strain YPH499 and *irc22Δ*. Ub-Leu-β-gal [48] was expressed in yeast under the control of the *GAL1* promoter. Cells were collected by centrifugation and transferred to galactose medium lacking uracil. The incubation was continued for 2 h at 30 °C to induce Ub-Leu-β-gal, and then dextrose and cycloheximide were added to a final concentration of 2% and 0.5 μg/mL, respectively. For over-expression of GST-Pth2 in Ub-Leu-β-gal-expressing cells, both plasmids carrying Ub-Leu-β-gal or pEG(KG)-*PTH2* were transformed into YPH499 cells. Cell extracts were prepared [49], followed by immunoblotting with anti-β-gal antibody. Half-life of Leu-β-gal degradation was quantified by a plot each showing % remaining *versus* time.

### 2.5. Growth Sensitivity to Salt Stress, UV Irradiation and Oxidative Stress

Yeast strains were cultured in YPD or selective medium at 30 °C for 14–16 h, and their cell densities were adjusted to OD_600_ = 1.0. Fivefold serial dilutions were prepared in sterile water, and 10 μL was spotted on YPD plates supplemented with various concentrations of NaCl, CaCl_2_, or MgCl_2_. Yeast cells on YPD plates were exposed to UV irradiation (50 or 100 J/m^2^) and H_2_O_2_ (0.5 or 1 mM). The sensitivity to salt stress, UV irradiation, and oxidative stress was determined after 2–4 days of growth at 30 °C or 37 °C. 

### 2.6. Antibodies

**Figure 1:**
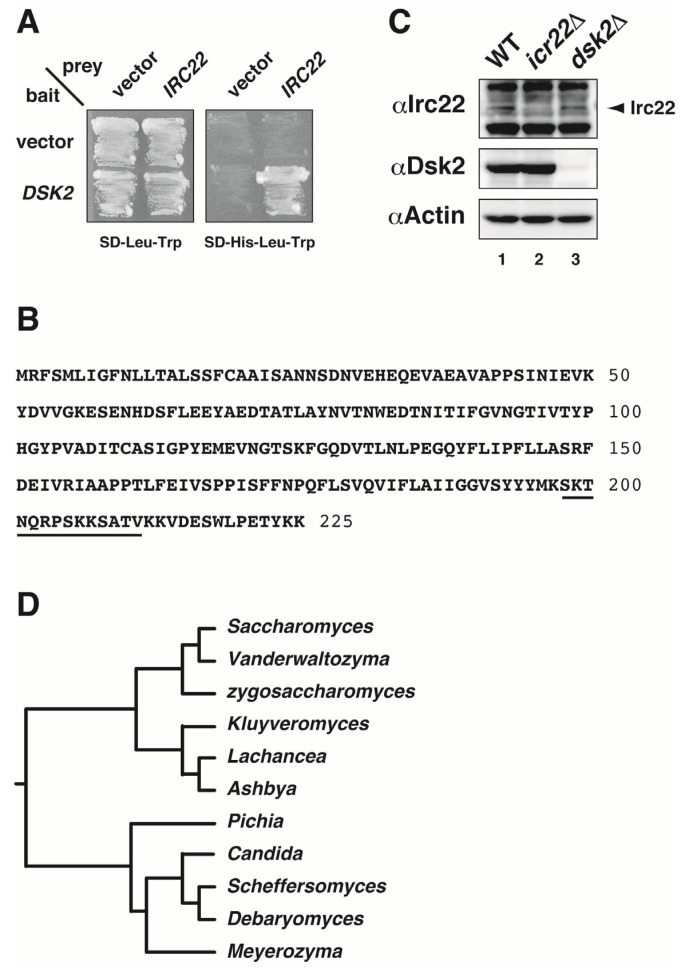
Identification of Irc22 in *Saccharomyces cerevisiae*. (**A**) Interaction of *IRC22* with *DSK2* in a yeast two-hybrid assay. The interaction between *IRC22* and *DSK2* was tested by histidine-prototrophic growth, using *DSK2* as bait and *IRC22* as prey. (**B**) Deduced amino acid sequence of Irc22. The deduced amino acid sequence of Irc22 is shown, and amino acid numbers are indicated at the right of the sequence. The amino acid sequence using the production of peptide antibody is underlined. (**C**) Immunoblotting of Irc22 protein. Cell extracts of wild-type strain (Lane 1), *irc22Δ* (Lane 2), or *dsk2Δ* (Lane 3) was immunoblotted with anti-Irc22 peptide antibody (top). An apparent molecular mass of Irc22 on SDS gels was estimated as ~34 kDa. Dsk2 and actin was detected as a positive (middle) and loading control (bottom), respectively. (**D**) *IRC22* homolog found in ascomycetous yeasts. Homologs of *IRC22/YEL001C* were searched using the database of Orthologous Groups (OrthoDB) and *Saccharomyces* genome database (SGD). On the basis of nucleotide sequence data from both databases, *IRC22* homologs were present among Saccharomycetes in Ascomycota.

The following antibodies were used: anti-Dsk2 [8], anti-GST and anti-actin from Santa Cruz Biotechnology (Santa Cruz, CA, USA), anti-T7 and anti-β-gal (Promega, WI, USA), anti-polyubiquitin (FK1, Nippon Bio-Test Laboratories, Tokyo, Japan). Anti-Irc22 polyclonal antiserum was raised in rabbits against synthetic polypeptides corresponding to the amino acid sequence SKTNQRPSKKSATV (positions 198-211) of Irc22 (see an underline in Figure 1B) (Operon Biotechnologies, Tokyo, Japan).

## 3. Results and Discussion

### 3.1. Identification of Irc22 as a Novel Dsk2-Interacting Protein in Yeast

Budding yeast Dsk2 is a UBL-UBA protein that participates in the ubiquitin-proteasome pathway. We screened for proteins that interacted with Dsk2 in a two-hybrid system. Among 1.5 × 10^5^ transformants from an *S. cerevisiae* cDNA library, three clones strongly interacted with Dsk2 (Figure 1A). All three clones encoded 225 amino acids with a calculated molecular weight of 25 kDa (Figure 1B), and the locus was identified as *YEL001C* on yeast chromosome V [50]. The *YEL001C* locus encodes *IRC22* (provisional description in *Saccharomyces* genome database (SGD)), the function of which is unknown [51]. Immunoblotting by anti-Irc22 antibody with yeast cell extracts showed that the Irc22 protein was detected in wild-type cells (Figure 1C, Lane 1) with an apparent molecular mass of ~34 kDa, but not detected in *irc22Δ* (lane 2). Based on two databases of SGD and Orthologous Groups (OrthoDB) [50,52], we searched the nucleotide sequence of the *IRC22* homolog and found that its homologs were conserved among ascomycetous yeasts (Figure 1D). *IRC22/YEL001C* is a non-essential gene for yeast growth [50] (see also Figure 4).

### 3.2. Irc22 Interacts with Dsk2 But Not with Rad23 and Ddi1

**Figure 2:**
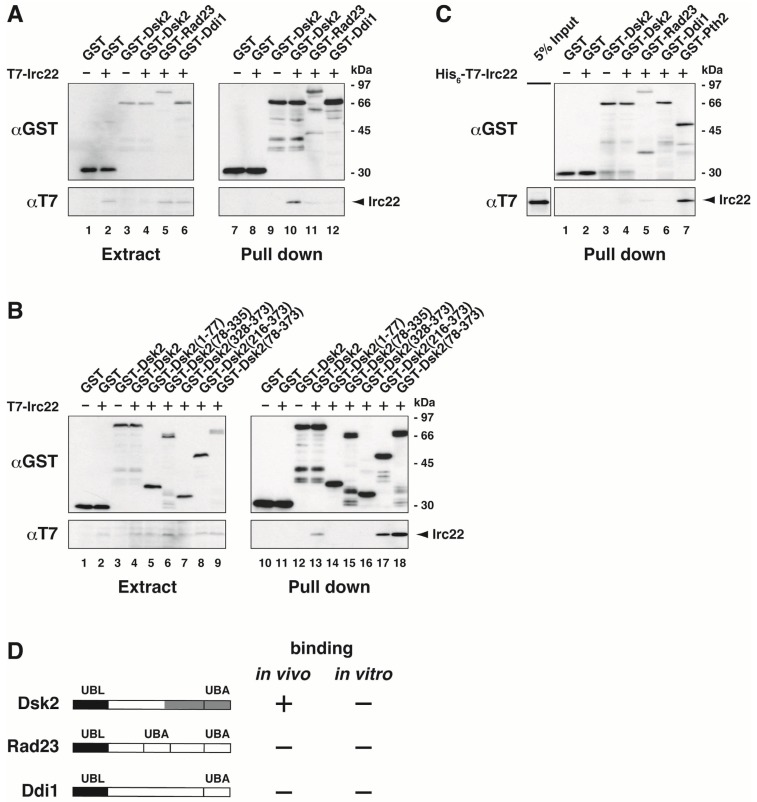
Irc22 interacts with Dsk2 but not Rad23 and Ddi1. (**A**) Interaction of Irc22 with Dsk2 *in vivo*. T7-Irc22 was co-expressed with GST-Dsk2, GST-Rad23, GST-Ddi1 or GST alone in wild-type yeast. The extracts (Lanes 1–6) and the GST-precipitates (Lanes 7–12) were immunoblotted as indicated. (**B**) The C-terminal UBA domain and the middle stretch of Dsk2 are required for its interaction with Irc22 *in vivo*. A series of truncated mutants of Dsk2 were constructed as indicated. T7-Irc22 was co-expressed with Dsk2 mutants in wild-type yeast, and the cell extracts (Lanes 1–9) were pulled down with glutathione-Sepharose 4B followed by immunoblotting with the indicated antibodies (Lanes 10-18). (**C**) Binding assay between Irc22 and UBL-UBA proteins *in vitro*. The binding of Irc22 to Dsk2, Rad23, Ddi1, or Pth2 (see Discussion) was tested *in vitro*. His_6_-T7-Irc22 was mixed with GST-Dsk2, GST-Rad23, GST-Ddi1, GST-Pth2, or GST alone in lysis buffer and incubated with glutathione-Sepharose 4B. The bead-bound materials (Lanes 1–7) were immunoblotted with the indicated antibodies. (**D**) Summary of Irc22 interactions *in vivo* and *in vitro*. Binding of Irc22 to Dsk2, Rad23, and Ddi1 *in vivo* and *in vitro* are summarized. Interaction sites of Irc22 are shaded in the middle stretch and the UBA domain of Dsk2 drawing.

We tested for an interaction between Irc22 and Dsk2 in yeast (Figure 2A). T7-Irc22 was co-expressed with GST-Dsk2 in yeast cells, and *in vivo* binding was tested using a GST pull-down assay followed by immunoblotting with anti-T7 antibody. T7-Irc22 co-precipitated with GST-Dsk2 (Lane 10). The binding of Irc22 with two other UBL-UBA proteins, Rad23 and Ddi1, was tested similarly. In contrast to Dsk2, T7-Irc22 did not co-precipitate with GST-Rad23 (Lane 11) and GST-Ddi1 (Lane 12). 

Next we investigated which domain of Dsk2 is required for binding to Irc22. A series of deletion mutants of Dsk2 was constructed (Figure 2B), and their binding to Irc22 was tested in yeast. The binding assay was carried out as in Figure 2A. Irc22 bound to 216-373 (Lane 17) and 78-373 (Lane 18), as well as full-length (1-373) Dsk2 (Lane 13), all of which contain the UBA domain. However, Irc22 did not bind to the UBA domain alone (328-373) (Lane 16). Also, Irc22 did not bind to the middle stretch alone (78-335) (Lane 15). Therefore, the UBA domain and the middle stretch of Dsk2 are both required for the binding with Irc22 *in vivo*. The reason why Irc22 selectively binds to Dsk2 could be because the middle stretch of the UBL-UBA proteins is not conserved between Dsk2, Rad23, and Ddi1. Compared with full-length Dsk2, Irc22 strongly interacted with the UBL domain-deleted fragment (Lanes 17 and 18), and the UBL domain alone was not required for the Irc22-Dsk2 interaction (Lane 14), indicating that the Irc22-Dsk2 interaction was partially inhibited by the interaction between the UBL and UBA domains. 

We also tested for direct binding *in vitro* between Irc22 and the UBL-UBA proteins (Figure 2C). His_6_-T7-Irc22 was mixed with GST-Dsk2, GST-Rad23, and GST-Ddi1 and incubated with glutathione-Sepharose 4B. The bead-bound materials were immunoblotted with anti-T7 antibody. Figure 2C shows that His_6_-T7-Irc22 does not bind GST-Dsk2, GST-Rad23, and GST-Ddi1 *in vitro* (Lanes 4, 5, and 6). Therefore, the binding of Irc22 to Dsk2 *in vivo* (Figure 2A) is indirect, which suggests that the Irc22-Dsk2 interaction may be mediated by unknown protein factor(s). The binding of Irc22 to UBL-UBA proteins *in vivo* and *in vitro* is summarized in Figure 2D.

### 3.3. Irc22 Affects the Ubiquitin-Dependent Degradation Pathway in Yeast

**Figure 3:**
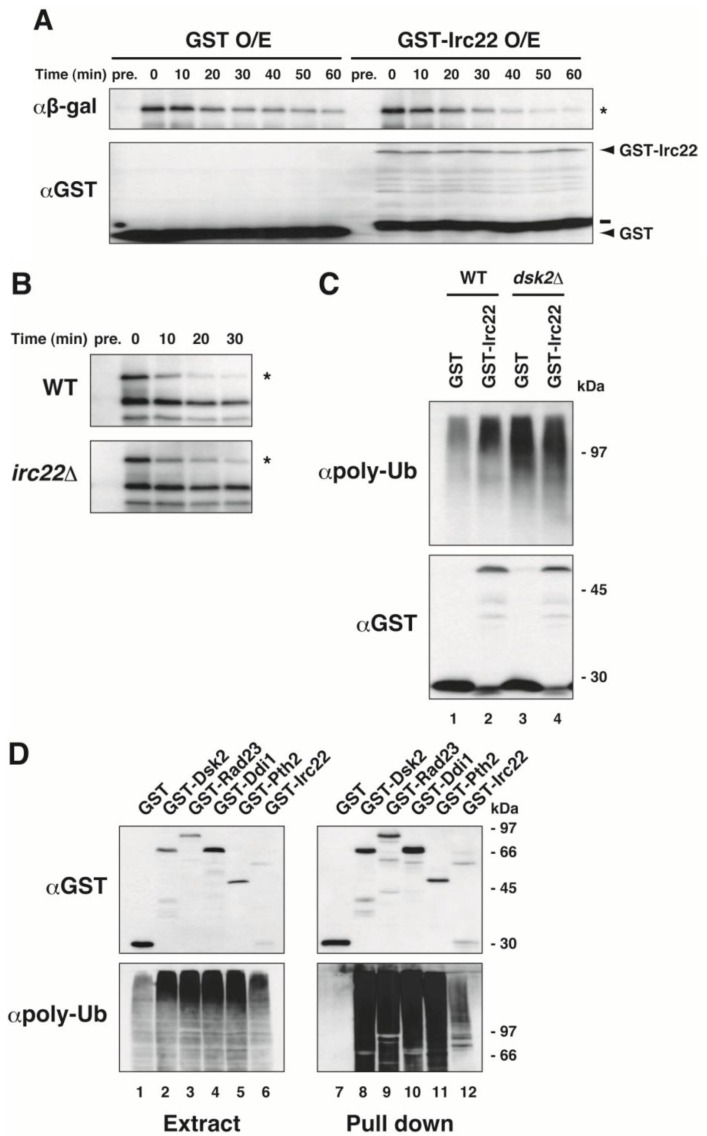
Irc22 affects the ubiquitin-dependent degradation pathway. (**A**) Acceleration of protein degradation in Irc22-overexpressing yeast cells. The N-end rule substrates Leu-β-gal and GST-Irc22 were co-expressed with galactose induction in wild-type yeast cells. After the induction was terminated, cell samples were collected at the indicated times, and the degradation of Leu-β-gal was assessed by immunoblotting with anti-β-gal and anti-GST antibodies. Pre.; before induction. An asterisk indicates Leu-β-gal. A bold bar denotes a proteolytic product of GST-Irc22. (**B**) Delay of protein degradation induced by *IRC22* disruption. Leu-β-gal was induced with galactose in either *irc22Δ* or wild-type cells. After the induction was terminated, cells were sampled at the indicated times, and Leu-β-gal degradation was followed by immunoblotting with anti-β-gal antibody. Pre.; before induction. Asterisks indicate Leu-β-gal. (**C**) Effect of Irc22 over-expression on polyubiquitinated proteins in yeast cells. GST-Irc22 was over-expressed in wild-type (Lane 2) and *dsk2Δ* (Lane 4) strains. The cell extracts were immunoblotted by anti-polyubiquitin and anti-GST antibodies. (**D**) *In vivo* binding of Irc22 to polyubiquitinated proteins in yeast cells. GST-Dsk2, GST-Rad23, GST-Ddi1, GST-Pth2, GST-Irc22, or GST alone was expressed in wild-type cells, and the cell extracts (Lanes 1–6) and GST pull downs (Lanes 7–12) were immunoblotted by anti-polyubiquitin and anti-GST antibodies.

We examined whether *IRC22* affects the ubiquitin-dependent protein degradation pathway (Figure 3A and B). The N-end rule substrate Leu-β-gal was used as a model substrate for the ubiquitin-proteasome pathway. Leu-β-gal was expressed by galactose induction in wild-type and *irc22Δ* strains. After the induction was shut-off by the addition of glucose, the amount of Leu-β-gal was followed in the indicated time and measured by immunoblotting with anti-β-gal antibody. Figure 3A shows that the over-expression of Irc22 in wild-type cells accelerated the degradation of Leu-β-gal. An estimated half-life of Leu-β-gal degradation was ~11 min in the presence of Irc22 and ~30 min in the absence of Irc22. In contrast, the degradation of Leu-β-gal was mildly slowed by disruption of *IRC22* compared with that of wild-type cells (Figure 3B). Half-life was ~10 min in *irc22Δ* and ~8 min in wild-type strains. The quantification of the experiments in Figure 3A and B is shown in Supplementary Data. Therefore, Irc22 positively affects the ubiquitin-dependent protein degradation.

We also tested the effect of Irc22 on polyubiquitinated proteins in yeast. GST-Irc22 was expressed in cells, and the cell extracts were immunoblotted with anti-polyubiquitin antibody (Figure 3C). Irc22 over-expression in the wild-type strain caused an accumulation of polyubiquitinated proteins in cells (Lane 2), although the effect of Irc22 on protein degradation seems to be relatively low. However, Irc22 over-expression in *dsk2Δ* did not exhibit a relative increase of polyubiquitinated proteins (compare Lanes 3 and 4). These results suggest that Dsk2 is involved in an accumulation of polyubiquitinated proteins that is caused by Irc22. 

We further tested the binding of Irc22 to polyubiquitinated proteins *in vivo* and *in vitro*. GST-Irc22 was expressed in cells and precipitated with glutathione-Sepharose 4B followed by immunoblotting with anti-polyubiquitin antibody (Figure 3D). GST-Irc22 bound weakly to polyubiquitinated proteins in cells (Lane 12). Compared with the strong binding of UBL-UBA protein Dsk2, Rad23, Ddi1 and Pth2 to polyubiquitinated proteins, binding of Irc22 to polyuibiquitinated proteins seems to be relatively weak. Irc22 alone did not bind directly to polyubiquitin chains *in vitro* (data not shown). 

### 3.4. IRC22 is Involved in Salt Stress Responses in Yeast

**Figure 4:**
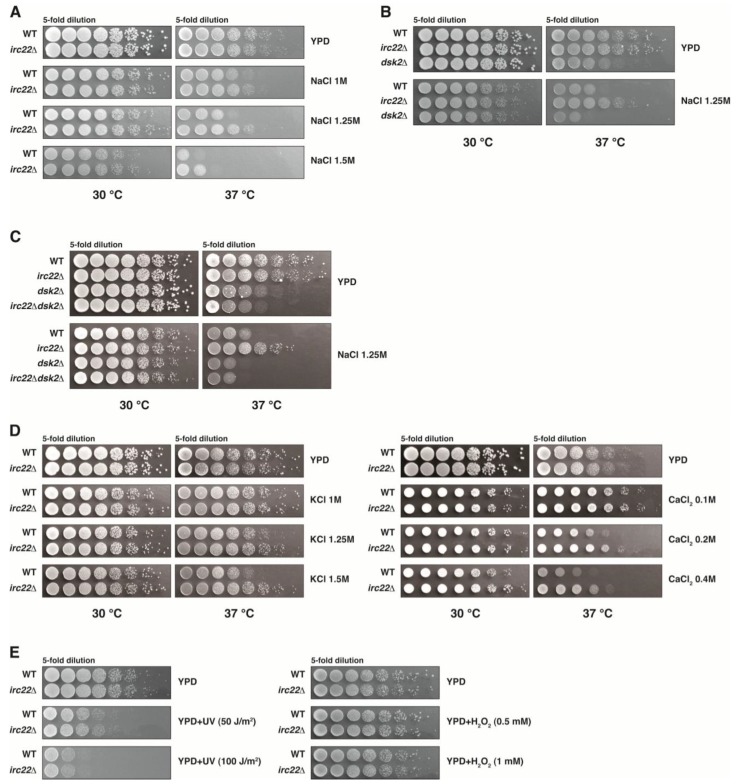
Dsk2 and Irc22 are involved in salt stress tolerance in yeast. (**A**) Growth tolerance to NaCl stress induced by *IRC22* disruption. Wild-type and *irc22Δ* cells were grown in YPD medium and spotted onto YPD plates containing 1.0–1.5 M NaCl at 30 °C or 37 °C in fivefold serial dilutions. (**B**) Growth sensitivity to NaCl stress induced by *DSK2* disruption. Wild-type, *irc22Δ* and *dsk2Δ* cells were grown in YPD medium and spotted onto YPD plates containing 1.25 M NaCl at 30 °C or 37 °C in 5-fold serial dilutions. (**C**) Disappearance of salt tolerance by *irc22Δdsk2Δ* double disruption. Wild-type, *irc22Δ, dsk2Δ*) and *irc22Δdsk2Δ* cells were grown in YPD medium and spotted onto YPD plates containing 1.25 M NaCl at 30 °C or 37 °C in 5-fold serial dilutions. (**D**) Growth tolerance to CaCl_2_ and KCl stresses induced by *IRC22* disruption. Wild-type and *irc22Δ* cells were grown in YPD medium and spotted onto YPD plates containing KCl (1.0–1.5 M; left panel) or CaCl_2_ (0.1–0.4 M; right panel) at 30 °C or 37 °C in 5-fold serial dilutions. (**E**) Disruption of *IRC22* affects neither DNA damage nor oxidative stresses. Wild-type and *irc22Δ* cells were grown in YPD medium. Left panel: cells were spotted onto YPD plates in fivefold serial dilutions and exposed to 50 J/m^2^ or 100 J/m^2^ UV irradiation at 30 °C. Right panel: cells were spotted onto YPD plates containing H_2_O_2_ (0. 5 mM and 1 mM) at 37 °C in fivefold serial dilutions.

The growth of the wild-type strain was inhibited by the addition of a high concentration of NaCl to the medium. Compared with wild-type cells, the disruption of *IRC22* conferred stress tolerance to cells exposed to high salt conditions (Figure 4A). Therefore, *IRC22* is negatively involved in salt tolerance. The salt-tolerant phenotype of *irc22Δ* was exhibited at a high temperature, 37 °C, but not at 30 °C. The salt tolerance that was observed in *irc22Δ* proved to be a temperature-sensitive phenotype. *IRC22* is a nonessential gene under optimal growth conditions, but *irc22Δ* has a detectable phenotype for growth under salt stress conditions.

We also found that *DSK2* becomes essential under salt stress conditions, although it is known to be nonessential for growth under optimal conditions [26]. We tested the effect of *DSK2* disruption on salt stress. Unlike *irc22Δ*, *dsk2Δ* exhibited salt sensitivity at 37 °C but not at 30 °C (Figure 4B). Therefore, Dsk2 is positively involved in salt stress tolerance. Similar to *irc22Δ*, the salt tolerance that was mediated by *dsk2Δ* also proved to be a temperature-sensitive phenotype.

As shown in Figure 4C, the salt tolerance that was observed in *irc22Δ* disappeared with the disruption of *DSK2* (*dsk2Δirc22Δ* bottom row): That is, the salt sensitivity at 37 °C was equivalent between *dsk2Δ* and *dsk2Δirc22Δ* in the presence of NaCl (Figure 4C, lower panel). Therefore, salt-tolerance by *IRC22* disruption depends upon *DSK2*, suggesting the hypothesis that Irc22 is negatively involved in salt stress tolerance via Dsk2 (see Discussion).

Salt tolerance by *IRC22* disruption was also observed in the presence of KCl or CaCl_2_ at 37 °C (Figure 4D). In contrast to the salt stress, the growth inhibition of *irc22Δ* with increasing dosages of UV irradiation and H_2_O_2_ was similar to that of the wild-type strain (Figure 4E). Thus, the disruption of *IRC22* did not affect any responses to DNA damage and oxidative stress compared with the wild-type strain. Overall, these data suggest that Irc22 participates in yeast salt stress tolerance depending on the Dsk2 function. Of the UBL-UBA proteins, Dsk2 seems to have distinct roles in salt stress tolerance involving Irc22 function.

### 3.5. Discussion

#### 3.5.1. Distinct Functions of the Middle Stretch of UBL-UBA Proteins

UBL-UBA proteins act as shuttle ubiquitin receptors in the ubiquitin-proteasome pathway. This delivery function of UBL-UBA proteins requires its N-terminal UBL domain and C-terminal UBA domain. Both UBL and UBA domains are well conserved among the UBL-UBA protein family including Dsk2, Rad23 and Ddi1. Thus, the role of UBL and UBA domains are basically the same among UBL-UBA proteins. In contrast, the middle stretch of UBL-UBA proteins is not conserved among the protein family. For instance, Rad23 possesses a second UBA domain and an XPC-binding domain in its middle stretch [53]. The middle stretch of Dsk2 contains repetitive STI1 motifs [39]. The middle stretch of Ddi1 shows high similarity to the aspartyl-protease domain [37]. Besides these identified domains, UBL-UBA proteins contain distinct sequences in the middle region of each protein. It becomes increasingly evident that UBL-UBA proteins are involved in not only protein degradation but also certain unique cellular functions. The middle stretch of each UBL-UBA protein seems to be closely related to its specific cellular functions, for example, as shown in this report in which the middle stretch of Dsk2 is required for its Irc22 interaction (Figure 2). 

#### 3.5.2. Irc22 Acts as a Negative Regulator of Dsk2 in Salt Tolerance

It has been not reported previously that Dsk2 is involved in salt stress. We show here that strains with a deletion of *DSK2* are sensitive to high salt concentrations; therefore, Dsk2 is required for salt tolerance (Figure 4B). In contrast, strains with a deletion of *IRC22* are tolerant to salt stress (Figure 4A). Moreover the salt tolerance observed in *irc22Δ* disappears with the deletion of *DSK2* (*dsk2Δirc22Δ*; Figure 4C, bottom row). Therefore, Irc22 suppresses Dsk2, which has a positive function in salt stress tolerance. Irc22 seems to negatively regulate salt stress tolerance. Because Irc22 does not interact with Rad23 and Ddi1 (Figure 2C), the function of Irc22 in salt stress would not involve Rad23 and Ddi1.

Based on the results above, we propose that Irc22 may act as a negative regulator of Dsk2 and regulates Dsk2 functions in salt stress tolerance via ubiquitin-dependent protein degradation. Dsk2 has already been shown to function positively in the ubiquitin-dependent degradation [8], and we suspect that Dsk2 via the ubiquitin-dependent degradation would be positively involved in salt stress tolerance. In high salt conditions, therefore, a loss of Dsk2 causes decreased ubiquitin-dependent degradation and results in salt sensitivity, and a loss of Irc22 causes increased Dsk2 function and results in salt tolerance because Irc22 represses Dsk2 (Figure 4). Irc22 over-expression leads to a slightly decreased salt tolerance (our preliminary result). 

In contrast to the negative involvement of Irc22 in salt stress, Irc22 positively affects protein degradation. As shown in Figure 3, ubiquitin-dependent degradation tends to be delayed by Irc22 deletion (Figure 3B) and accelerated by over-expression of Irc22 (Figure 3A). In normal growth conditions, therefore, Irc22 participates positively in the ubiquitin-proteasome pathway. Because Irc22 apparently behaves as a positive regulator in the protein degradation, we suspect that some negative regulator(s) would mediate the Irc22-Dsk2 interaction. Consequently, as seen in Figure 3, ubiquitin-dependent degradation would be accelerated by Irc22 over-expression and be retarded by a loss of Irc22. We have found that Dsk2 interacts with Irc22 *in vivo* but not *in vitro* (Figure 2), indicating that Irc22 interacts with Dsk2 via some mediator(s). Because Irc22 binds directly to Pth2 (Figure 2C, Lane 7), Pth2 may be a candidate for a putative mediator that is involved in the Irc22-Dsk2 interaction. Pth2 has been shown to bind Dsk2 and inhibit protein degradation [21]. Our simple expectation is that the Irc22-Dsk2 interaction via such mediator(s) could contribute to the ubiquitin-proteasome degradation pathway. The Irc22-Dsk2 interaction in high salt conditions might pass over the mediator(s) at high temperature.

We also prefer to speculate that the Irc22-Dsk2 interaction would be mediated through the use of different domains of Dsk2. For example, the N-terminal UBL and C-terminal UBA domains of Dsk2 are required for protein degradation, mediating Lys48-linked chains. In contrast, the middle stretch of Dsk2 cooperates with the C-terminal UBA domain and could participate in salt stress tolerance, mediating other linkage types like Lys63-linked chains [1,2,54,55]. In the presence or the absence of salt stress, Irc22 may modulate the Dsk2 function using different ubiquitin chains.

#### 3.5.3. Possible Link between the UBL-UBA Protein Dsk2 and Salt Stress Tolerance in Yeast

Recently, several lines of evidence suggest that a ubiquitin-dependent degradation links directly to salt stress responses in plants. For instance, RPN10-mediated degradation plays a role in salt stress tolerance [41]. The E3 ubiquitin ligase is a positive regulator of high salt stress [43]. UBC32 functions in brassinosteroid-mediated salt stress tolerance [42]. ER-associated degradation is necessary for plant salt tolerance [44]. Moreover, abnormal proteins damaged by high salt would be removed from cells by a proper action of a protein degradation pathway. It is thus probable that the salt-stress phenotypes observed in *irc22Δ* and *dsk2Δ* could be due to altered ubiquitin-dependent degradation. 

Intriguingly, the salt-stress phenotypes of *irc22Δ* and *dsk2Δ* were observed at 37 °C but not at 30 °C (Figure 4). A strain with deletions of both *DSK2* and *RAD23* is temperature-sensitive for growth [26,27]. Similarly, a temperature-sensitive growth inhibition was observed in inositol auxotrophy phenotype in yeast membrane phospholipids mutants [56,57]. Inositol phospholipids have crucial roles in membrane trafficking, and inositol polyphosphates are essential for the proper responses to salt stress [58,59]. *DSK2* and *RAD23*, including several components of the proteasome function, were identified as non-essential genes that are necessary for growth in the absence of inositol [60]. Considering such circumstantial evidence, the Irc22-Dsk2 interaction could modulate the capacity of ubiquitin-dependent degradation pathway and thereby lead to an alteration of the composition and properties of membrane protein and/or membrane lipid cooperating with salt stress and osmotic stress. In fact, a ubiquitin-dependent degradation is known to play a crucial role in the degradation of membrane proteins for proper cellular functions [18,42,61,62]. It is also possible that the Irc22-Dsk2 interaction affects altered degradation of membrane protein(s) involved in membrane ion flux. At present, however, it would be premature to specify which actions for salt stress involve the Irc22-Dsk2 interaction. We can imagine in yeast that the UBL-UBA protein Dsk2, together with Irc22, would participate in salt stress tolerance through the process of ubiquitin-dependent degradation. 

In addition, Irc22 is predicted to localize to the ER membrane [51], and *IRC22* has been identified as one of the genes affecting vacuole membrane fragmentation [63]. It is intriguing, however, that *IRC22* homologs are present only among Saccharomycetes that belong to Ascomycota (Figure 1D) but not found in other species [50,52]. This implies either that Irc22 may play certain distinct functions in budding yeast or that functional homologs of Irc22 are present in other species. Further studies on these aspects are needed to clarify the unique and precise roles of Irc22 together with Dsk2 in salt stress tolerance.

## 4. Conclusions

A novel Dsk2-interacting protein Irc22 was identified in yeast. Irc22 interacts with Dsk2 but not with Rad23 and Ddi1. It has already been established that Dsk2 participates in the delivery of ubiquitinated proteins to the proteasome in the ubiquitin-dependent degradation pathway. In addition to known functions of Dsk2 in intracellular protein degradation, this report presents the first evidence that the UBL-UBA protein Dsk2, together with Irc22, is involved in salt stress tolerance.

## Supplementary Files

Supplementary File 1 Supplementary Materials (PDF, 109 KB)
