# Supplementary material for: Yeast Irc22 Is a Novel Dsk2-Interacting Protein that Is Involved in Salt Tolerance
Source: Cells. 2014 Mar 27;3(2):180–98. doi: 10.3390/cells3020180 (PMC4092868; doi:10.3390/cells3020180)
Supplement: Supplementary File 1 — Supplementary Materials (PDF, 109 KB) [file cells-03-00180-s001.pdf]

Supplementary Materials

Figure S1. Degradation of N-end rule substrate Leu-β-gal by overexpression and deletion of *IRC22*.

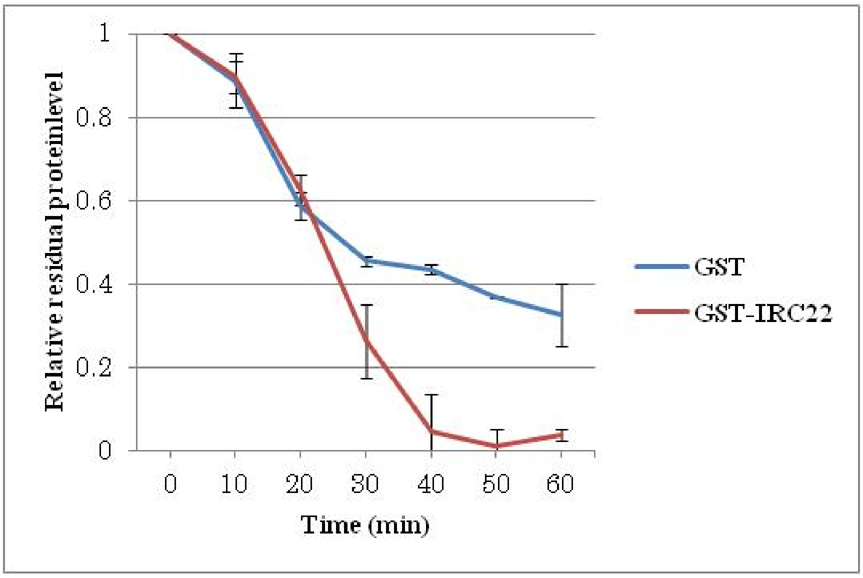

A

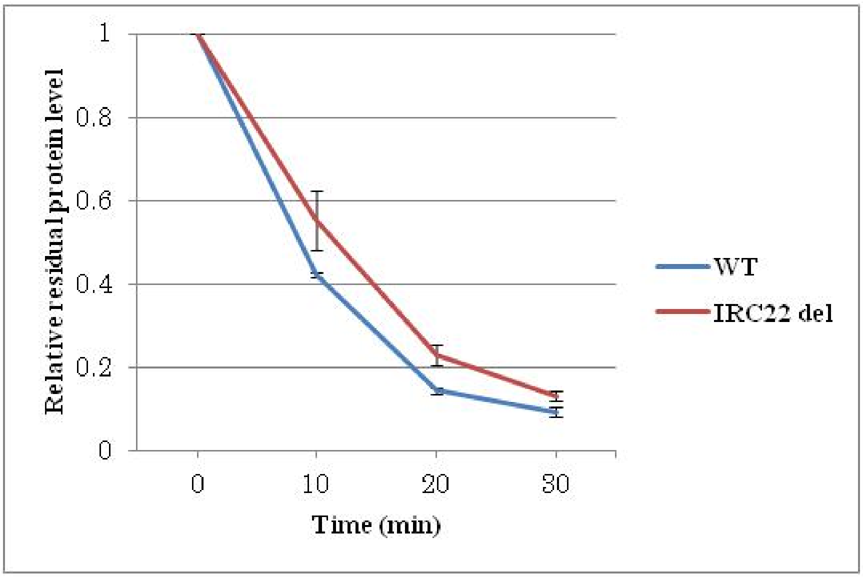

B

Based on the data in *IRC22* overexpression (Figure 3A) and in *irc22Δ* (Figure 3B), the degradation of Leu-β-gal was quantified and plotted over time by using ImageJ. The mean values (% inhibition) were averaged by two or three independent experiments. (A) *IRC22* overexpression; (B) *irc22Δ*.
